# The Use of Cross-Linked Hyaluronic Acid in Non-surgical Rhinoplasty Using Italian Technique

**DOI:** 10.1007/s00266-024-04197-6

**Published:** 2024-06-28

**Authors:** Antonio Scarano, Andrea Sbarbati, Domenico Amuso, Roberto Amore, Segio Rexhep Tari, Iris Alla

**Affiliations:** 1 Department of Innovative Technologies in Medicine and Dentistry, University of Chieti-Pescara, Via Dei Vestini 31, 66100 Chieti, Italy; 2 Department of Neurosciences, School of Medicine, Biomedicine and Movement Sciences, Anatomy and Histology Section, University of Verona, Verona, Italy; 3 Department of Innovative Technologies in Medicine and Dentistry, University of Chieti-Pescara, Chieti, Italy; 4 Department of Innovative Technologies in Medicine and Dentistry, University of Chieti-Pescara, Chieti, Italy

**Keywords:** Rhinoplasty, Hyaluronic acid, Filler, Aesthetic, Non-surgical rhinoplasty, Nose reshaping, Rhinofiller

## Abstract

**Introduction:**

Projection and upper rotation to the tip is fundamental in the nasal rejuvenation, as a matter of fact the tip is the most important and has strongly effects on the improve appearance and quality of life. The aim of the present study was to evaluate reshaping the tip of the nose by cross-linked hyaluronic acid using Italian technique.

**Methods:**

In a period between November 2019 and 2023, a total of one hundred and forty healthy, 95 females and 45 man patients, were performed with a mean age 44±5 (age range: 31–52 years old) affected by tip of congenital (22) or ageing nose hypotonia (118), and reduced volume that need of an elevation of the nose tip. The anatomic markers have been considered for the anthropometric measurements after the filler rhinoplasty. Two infiltrations were performed, one in the infiltration into the antero-caudal access over the columella produce upward rotation of the tip of the nose and second infiltration into the antero-superior access produce the projection of the tip of the nose. Medical device used in the study was Neofound STRUCT LIDO (LOVE COSMEDICAL srls–Via Toniolo 9, 57022 Castagneto Carducci, ITALY) containing sodium hyaluronate/hyaluronic acid high molecular weight (1.500<HA<2.000 KDA) 24%, sodium hyaluronate/hyaluronic acid low molecular weight (155<HA<230 KDA) 9%, niacinamide, glycine, proline, BDDE, and lidocaine chlorhydrate 3%.

**Results:**

The effect on the upward rotation of the tip nose was evaluated using Global Aesthetic Improvement Scale and morphometric evaluation. All the subjects showed at least 2–3 grade improvement in GAIS score after HA filler injection. The analysis of patient satisfaction after the last follow-up visits clearly demonstrated good results. A significant morphometric difference was detected comparing the T_0_ and T_90_ (*p*<0,0001), while no difference was present comparing T_90_ and T_180_ means (*p*=0.11).

The outcome of the present clinical study gives greater projection and upper rotation to the tip with great gratification of the patients and the surgeon. An augmentation of the tip nose with hyaluronic acid filler produces a rejuvenation of the nose area resulted in a more youthful appearance. No adverse event was observed. In 35 patients, additional HA infiltration had to be performed after 2 weeks.

**Conclusion:**

In conclusion, the Italian technique descripted in the present paper is safe, simply, and efficacious for rejuvenation of the nose, with elevated levels of patient satisfaction.

**Level of Evidence II:**

This journal requires that authors assign a level of evidence to each article. For a full description of these Evidence-Based Medicine ratings, please refer to the Table of Contents or the online Instructions to Authors www.springer.com/00266.

## Introduction

The nose plays a major role in facial aesthetics for its central location. The dorsum and the tip play an important role in the aesthetic the nose and face. During ageing, there is nasal tip, and cartilage ptosis may trigger that condition the aged appearance of the face. Projection and upper rotation to the tip is fundamental in the nasal rejuvenation, as a matter of fact the tip is the most important and has strongly effects on the improve appearance and quality of life. Noninvasive nasal reshaping procedure has increased in recent years. Rhinoplasty and rhinofiller are two techniques to improve the tip of the nose. Rhinoplasty is one of the most performed surgical treatments around the globe [1]. The first surgery date back to 1887, when the otolaryngologist John Orlando Roe described the first rhinoplastic procedure [2]. Since them, important advancements have been done thanks to the improved knowledge of the anatomical structures and related physiology.

Rhinoplasty could be performed due to different reasons: as a cosmetic treatment to pursue a desired aesthetic result; to recover or maintain the correct nasal function in case of reduced airflow caused by an obstructive process; and to heal a fracture after a collision, fall, or accident. Indeed, nasal fractures account for more than the 50% of facial fractures. A recent study evaluating a lapse of time from 2006 to 2014 and gathering data from the Nationwide Emergency Department Sample stated a total of 1,253,399.741 between open and closed nasal fractures, with an increasing trend in terms of costs for the Health Insurance System [3].

In despite of the great advances achieved to date, rhinoplasty remains the most complex surgical procedure among the plastic surgery treatments of choice. Moreover, as every surgical procedure, it is accompanied by several disadvantages, standing out breathing disturbances, atrophy, scars and fibrosis of the skin and soft tissues, infections and even postoperative deformities, depending on the surgeon and the circumstances nasal reshaping [4]. For all these reasons, non-surgical treatments are acquiring an increasing importance within the cosmetic field, preferring soft tissue fillers such as the rhinoplasty with liquid cartilage grafts, autologous fat, platelet-rich fibrin (PRF), and hyaluronic acid (HA). Today the most commonly used material is the uses hyaluronic acid (HA). All of these materials allow you to increase the volume of the nose; however, they have the disadvantage that they reabsorb, so the result lasts about 6 months. In fact, the American Aesthetic Society in their last Aesthetic Plastic Surgery National Databank Statistics from 2019 reported a reduction of the nose surgery in women (−10.7%) from 2015 to 2019 [5], a decrease that was as well reported by the British Analysis of BAAPS Audit 2020–2021 [6]. This is probably due to the inclusion of HA treatment as the preferred treatment for rhinoplasty instead of the most invasive surgical procedure.

By definition, hyaluronic acid is a biopolymer with a great stability, biocompatibility, and biodegradability [7], naturally present in the human and others animals body and concretely at the periphery and interfaces of elastin and collagen fibres, which has contributed to its always increasing role in cosmetics and nutricosmetics [8]. HA was used for rejuvenation of different facial areas, such as lips [9, 10], zygomatic area [11], eyebrow area [12], etc. HA filler for nose reshaping is a fast, effective, and safe treatment, with the injections being performed using small amounts of HA and a thin needle to stabilize and reshape the nose [13]. Numerous advantages are related to the use of HA fillers for rhinoplasty, as its less invasiveness, low procedure risks and post-treatment complications, faster recovery, temporary or reversible results, and the possibility to correct minor aesthetic defects, among others. However, HA use can also lead to complications such as infection; hypersensitivity; and vascular disorders. The ageing process of the face impacts the appearance the youthful appearance of men and women. The collapse of tip nose alters the facial morphology into one that is instantly recognizable as an aged face. The reshaping the tip of the nose with surgical and surgical procedure is a good treatment for rejuvenation of the face for increase the appearance of men and women.

The aim of the present study was to evaluate reshaping the tip of the nose by cross-linked hyaluronic acid using Italian technique [14].

## Materials and Methods

The present clinical study was based in a private practice in Chieti (Italy) and Janabelle Clinic Dubai (United Arab Emirates) in full accordance with ethical principles, including the World Medical Association Declaration of Helsinki and the additional requirements of Italian law and the United Arab Emirates. In a period between November 2019 and 2023, a total of one hundred and forty healthy, 95 females and 45 man patients, were performed with a mean age 44±5 (age range: 31–52 years old) affected by tip of congenital (22) or ageing nose hypotonia (118) and reduced volume that need of an elevation of the nose tip. All subjects signed an informed consent for the clinical study and were treated by rhinofiller procedure. Patient with active skin disease, irritation, history of autoimmune diseases, or inflammation in the target areas of injection were excluded. Also patient with poor general health, breastfeeding or pregnancy, known hypersensitivity or allergy to the treatment components, haemorrhagic diathesis, perforated nasal septum, severe functional deficits, and anticoagulant therapy and patient with previous filler treatments in the last months in the area were excluded. Also, the patients undergo rhinoplasty were excluded.

### Reshaping the Tip of the Nose Procedure

Medical device used in the study was Neofound STRUCT LIDO (LOVE COSMEDICAL srls–Via Toniolo 9, 57022 Castagneto Carducci, ITALY) containing sodium hyaluronate/hyaluronic acid high molecular weight (1.500<HA<2.000 KDA) 24%, sodium hyaluronate/hyaluronic acid low molecular weight (155<HA<230 KDA) 9%, niacinamide, glycine, proline, BDDE, and lidocaine chlorhydrate 3%. For reduce pain during infiltration of the filler, nose area was covered with an anaesthetic cream containing prilocaine and lidocaine (Emla, AstraZeneca, Sweden) for blocking nerve signals. The cream was applied 25–40 min before the treatment, removed with gauze, and the skin was disinfected with chlorhexidine 0.2% immediately for 2 min before the injections. The procedure requires only two deep boluses.

#### Step 1: Upper Rotation of the Tip

**Fig. 1:**
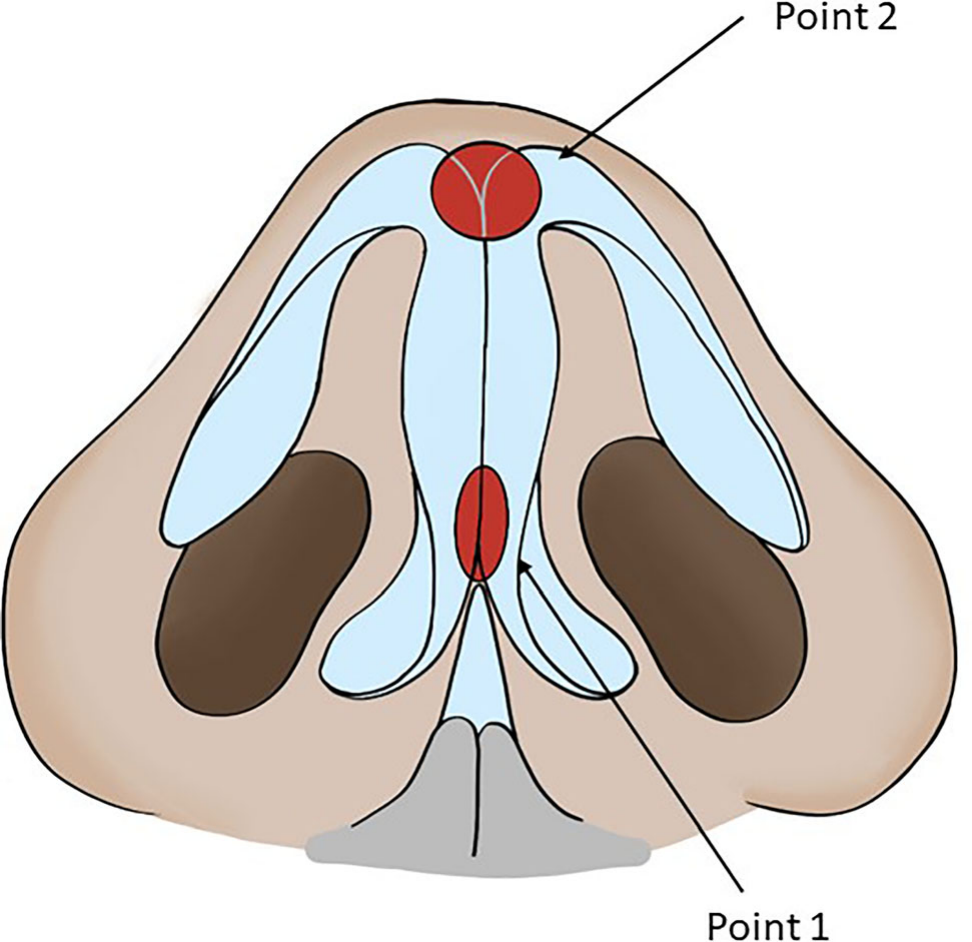
Point the infiltration into the antero-caudal access over the columella produce upward rotation of the tip of the nose Point 2—the infiltration into the antero-superior access produce the projection of the tip of the nose

**Fig. 2:**
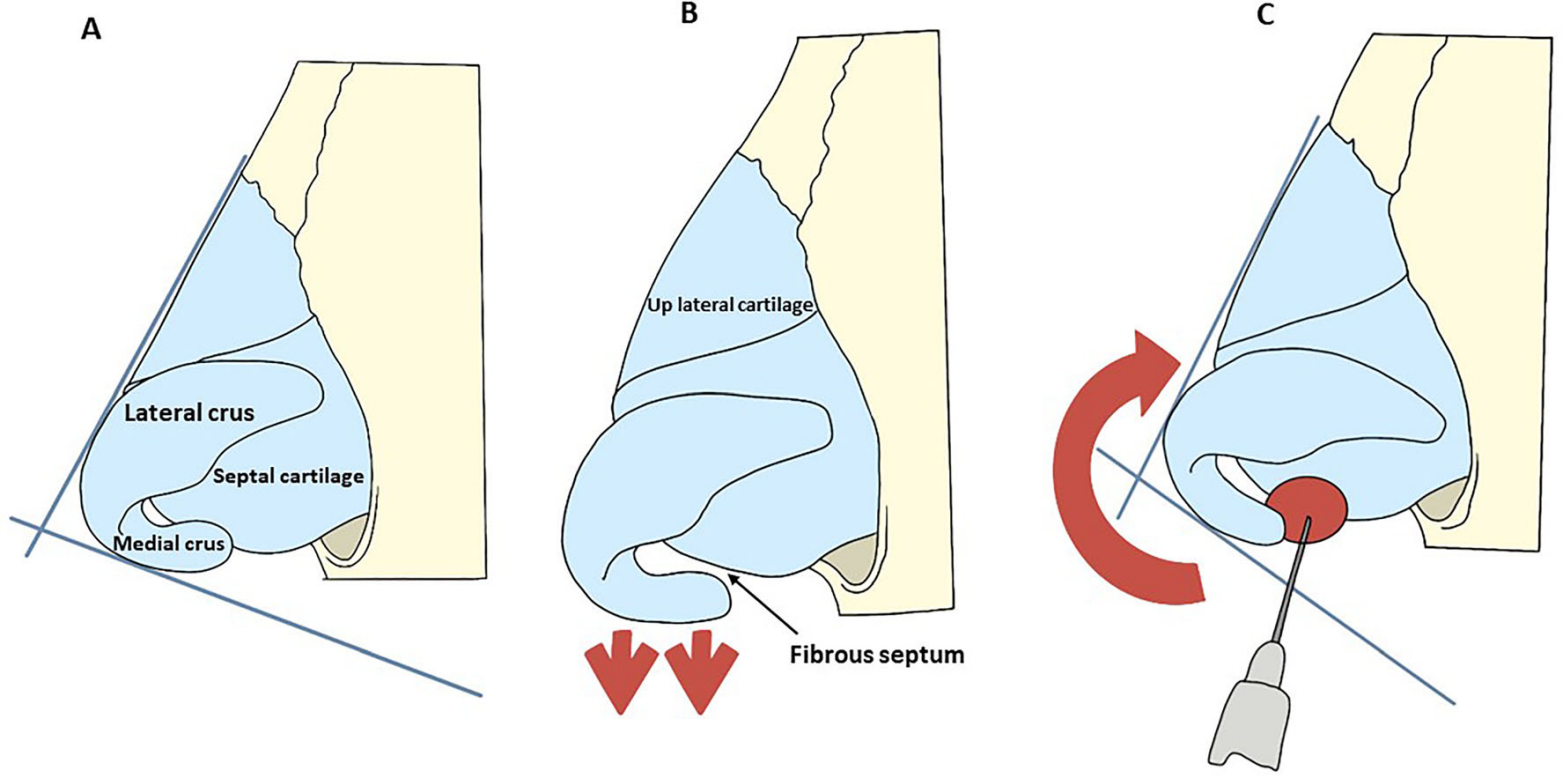
The figure shows that sequence shows step 1. **A**—The initial figure highlights a downward hypo-normorotated tip. **B**—With the first and second fingers of the free hand, it causes subluxes the wing cartilage, exposing the fibrous septum behind it and in front of the quadrant. **C**—A 0.2–0.3-ml bolus of hyaluronic acid that is released behind and in front of the quadrangular septum cartilage

With the first and second finger of the free hand, clamp the columella in order to palpate and perceive the two medial crura anteriorly and posteriorly and the cartilage of the septum (Fig. 1). Next, subluxate the medial crura by pulling them anteriorly in order to space the same crura from the quadrangular cartilage of the septum and stretch the fibrous septum, creating a space where the HA is to be injected. With the needle, enter the front face of the columella, passing between the medial crura to reach the fibrous septum and release a 0.1–0.3-mL bolus (average 0.22 mL) in the lower third of this space (Fig. 2). The released HA must be perceived between the fingertips. To ensure a safe releasing of the HA in the correct position, with the tip of the needle, before extruding the filler, support the anterior margin of the quadrangular cartilage of the septum and then retract a few millimetres (2–3 mm).

#### Step 2: Tip Projection

**Fig. 3:**
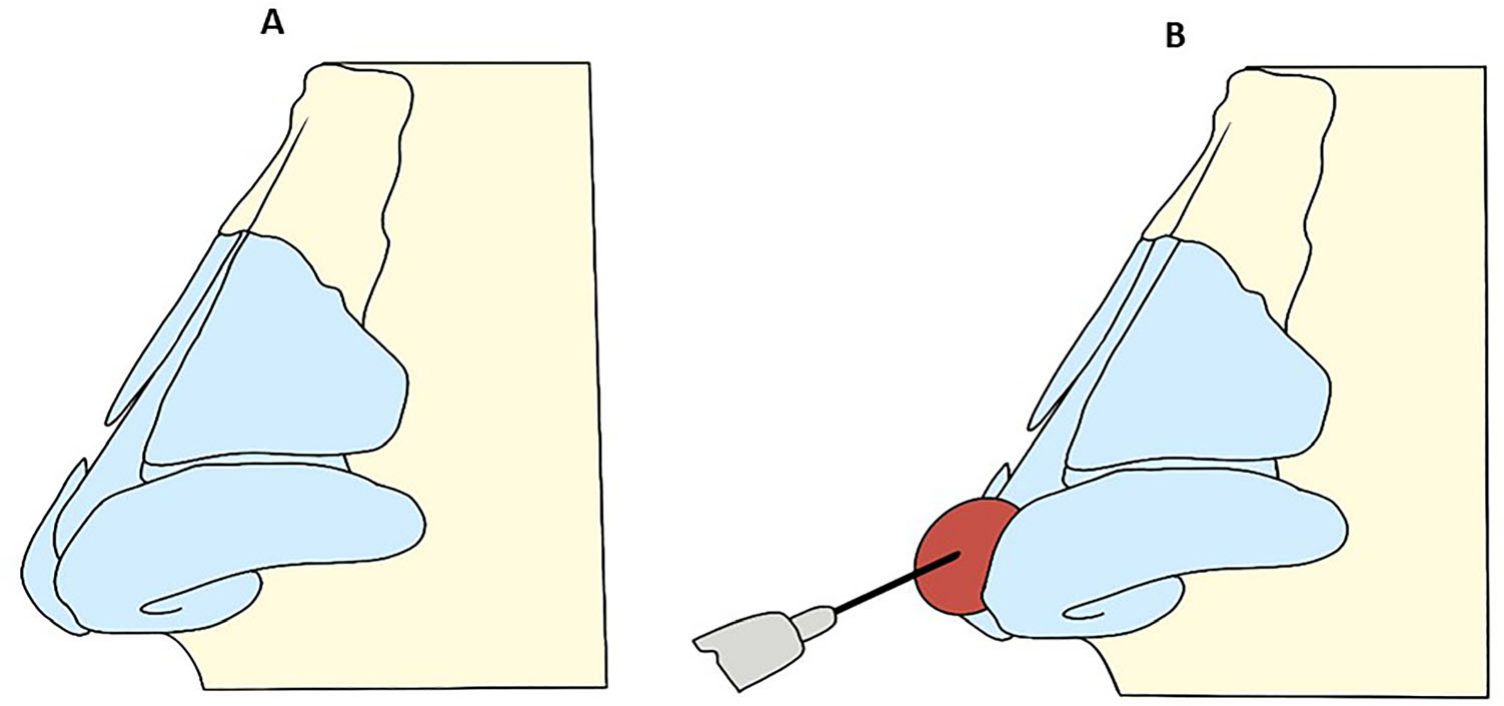
The figure shows that sequence shows step 2. **A**—The initial figure highlights a downward hypo-normoprojected tip. **B**—The bolus of 0.2–0.3 mL of hyaluronic acid (HA) into the antero-superior access produce the projection of the tip of the nose. The deep positioning of the HA prevents the occurrence of skin irregularities and conserves the vascular system, which is situated more superficially (in the SMAS)

The needle then enters anteriorly the interdomale space in the medial sagittal plane and then deeply releases a bolus of 0.1–0.3 mL (average 0.19 mL) of HA (Fig. 3) in order to increase the projection of the tip (Fig. 3). The needle 27G entered the skin at an angle of 80°–90° to the surface. The HA was be inoculated slowly, in a retrograde manner with a microbolus technique. The procedure follows a sequence: The first step is to treat the inter-columellar area; the second step is to treat the tip area. We fill until we obtain an upward rotation of the tip of the nose, usually 0.2 ml (average 0.19 mL) of HA is enough to achieve the result. No case was used the cannula. The remaining parts of the nose were left without any treatment. Among all the different molecules used as dermal filler agents, in the present investigation, we choice HA despite other filling agents were mandated by the fact because HA is reversible, and a specific antidote (hyaluronidase) is available, and because the authors have a great experience in terms of safety durability, and outcome. Five different aesthetic surgeons treated the patients. Patients are being pre-screened to apply intermittent local application of ice for 10 min and avoid massage the treated area. No oral cortisone or anti-inflammatories was prescribed. It has been advised to sleep supine position with the head upward and to drink a lot of water for 2–3 days after the treatment. The nose was photographed in frontal and oblique views with standardized zoom and automatic focus. Photos were taken before, immediately after the filler treatment, after 3 months and after 6 months for anthropometric evaluation.

Also the patient and the surgeon satisfaction were evaluated by means of a validated Global Aesthetic Improvement Scale (GAIS):

- Grade 5: Excellent (completely satisfied with the result)
- Grade 4: Very good (very satisfied with the result)
- Grade 3: Satisfactory (although a slight improvement is seen, an additional correction is required)
- Grade 2: Indifferent (no changes)
- Grade 1: Unsatisfied (the patient´s condition is worse than before the procedure)

Percentage change in nasal tip area and change in subjective patient scores were compared between before and after 3 months and 6 months after treatment.

### Morphometric Measurements

**Fig. 4:**
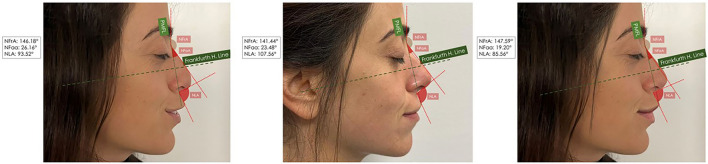
A significant difference NFaA was reported comparing the T_0_ and T_90_ (*p*<0,0001), and comparing T_90_ and T_180_ means (*p*<0,0001)

The following anatomic markers have been considered for the morphometric measurements after the filler rhinoplasty as described by Rho et al. [15]: prn: pronasale landmark; sn: subnasale landmark; pg: pogonion landmark; NFrA: the nasofrontal angle; NLA: the nasolabial angle; and NFaA: the nasofacial angle (Fig. 4). The measurement has been conducted at the baseline (T_0_), after 90 days (T_90_), and 180 days (T_180_) by two calibrated examiners. The concordance has been evaluated through the Cohen’s kappa (κ) and the Bland–Altman inter-operators agreement to test the bias. The study data have been evaluated by the statistical software package GraphPad Vers.9.0 (Prism, San Diego, CA, USA). The normality has been tested considering the Kolmogorov–Smirnov method, and the groups comparison has been evaluated through the Kruskal–Wallis test followed by the Dunn’s post hoc method.

### Statistical Analysis

The sample size to demonstrate the relative overall improvement after HA filler injection was calculated using a software for determining the number of patients to achieve statistical significance for validated Global Aesthetic Improvement Scale (GAIS). The planned enrolment was 140 subjects, presuming a drop-out rate of 10%. The sample size was calculated for dichotomous variables (yes/no effect) by the incidence effect (before treatment: 80% and after treatment: 20%); the alpha error was set at 0.001, and power was 95%. The optimal sample size for the evaluation was 58 patients. Numerical results are presented as the ±SD means of all the experiments. The study data were statistically analysed by the dedicated software package GraphPad 6 (Prism, San Diego, CA, USA). The normality distribution was tested by the Kolmogorov–Smirnov analysis. The Student's t-test was conducted for a statistically significant comparative evaluation between the study groups. The level of significance was set at *p*<0.05.

## Results

### Global Aesthetic Improvement Scale

No drop-out was recorded. The effect on the upward rotation of the tip nose was evaluated using Global Aesthetic Improvement Scale (GAIS). GAIS was evaluated after HA filler injection matched to before injection, by the subjects. During the follow-up, the comparative photographic documentation was taken. No erythema was present to a minimal extent immediately after filler treatment, and no oedema was recorded at day 1. The results immediately 3 and 6 months after the treatment showed a mean patient satisfaction score, respectively, 4.3±0.3 and 3.9±0.2, while the surgeon satisfaction index was 4.3±0.4 after the procedure and 3.9±0.2 at 6 months. A statistically significant difference was detected from before to 3 months after treatment (*p*<0.01) and after to 6 months (*p*<0.05). All the subjects showed at least 2–3 grade improvement in GAIS score after HA filler injection. The analysis of patient satisfaction after the last follow-up visits clearly demonstrated good results. There were no observed important adverse events such as outbreaks of ecchymosis, herpes, hyperpigmentation, hypopigmentation, itching, erythema, pain, infectious processes nor scarring, or allergy. In 25 patients, additional HA infiltration had to be performed after 2 weeks.

No statistical differences were detected between patients and surgeon Global Aesthetic Improvement Scale (GAIS) at baseline, 3 months after the treatment, and at 6 months.

Comparative analysis of photographic documentation showed that after 6 months, the volume obtained was reduced, but the patients were still satisfied with these results. Decrease in the increase in volume of the nose tip region was observed by only three patients, that might need another treatment. No new treatment was scheduled for asymmetry treatment during fullow-up. Macroscopically, a clinical effect with an appreciable change in tip nose was documented immediately after the procedure. Hyaluronic acid deposits were still visible at the 6 months follow-up. The good result remains stable at 6 months. After 6 months, a gradual return to baseline is observed and in a few case is necessary to repeat the procedure.

### Morphometric Measurements Results

**Fig. 5:**
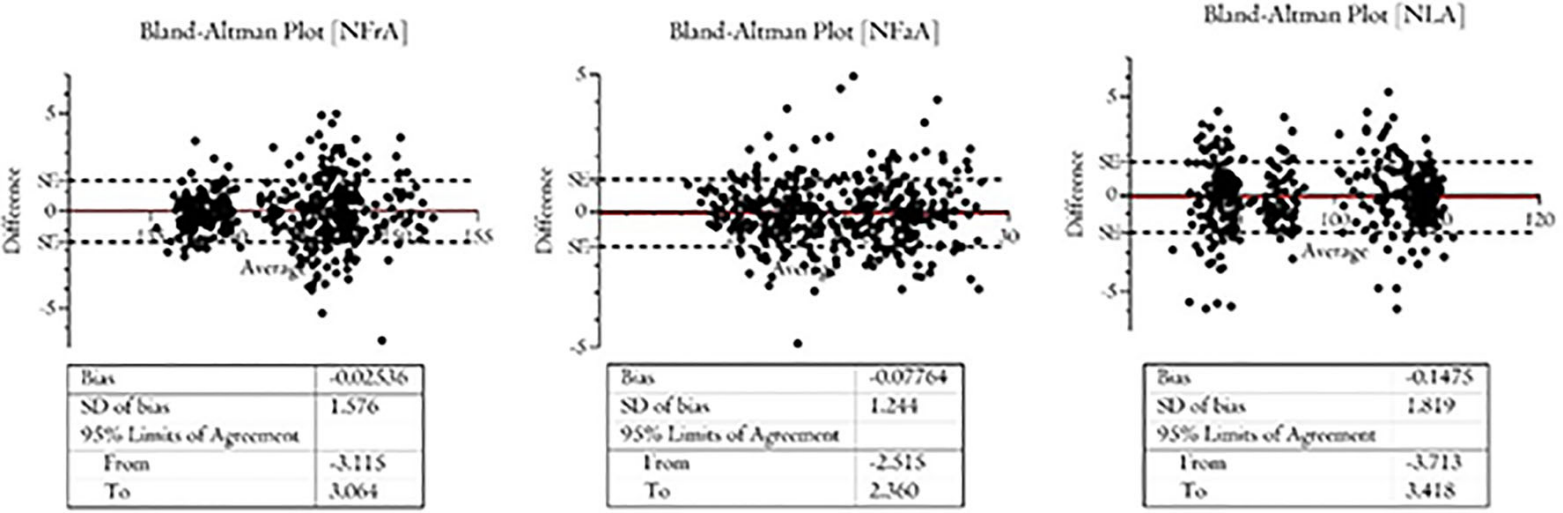
Bland–Altman plot comparison of the inter-class measurements

**Fig. 6:**
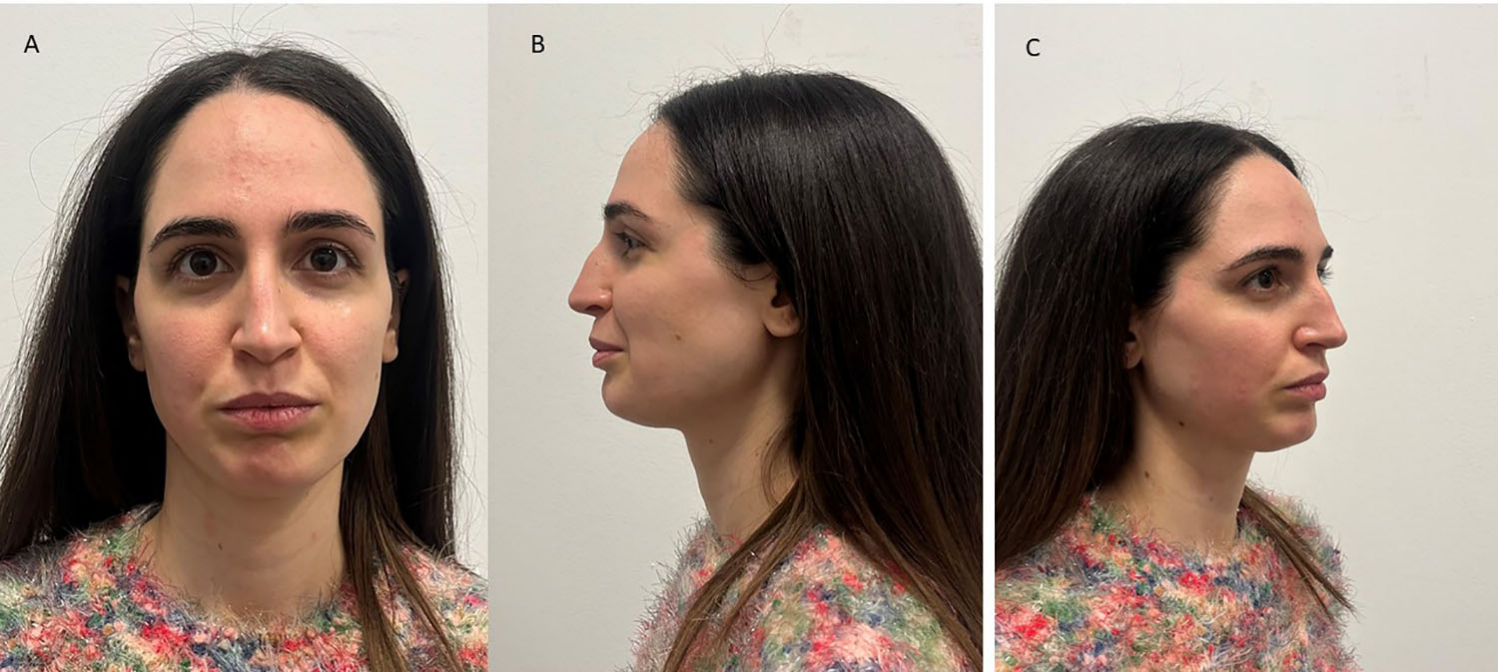
The initial state of pre-treatment. The patient shows a hypo-projected tip in frontal view (**A**), lateral view (**B**), and at 45 degrees (**C**)

**Fig. 7:**
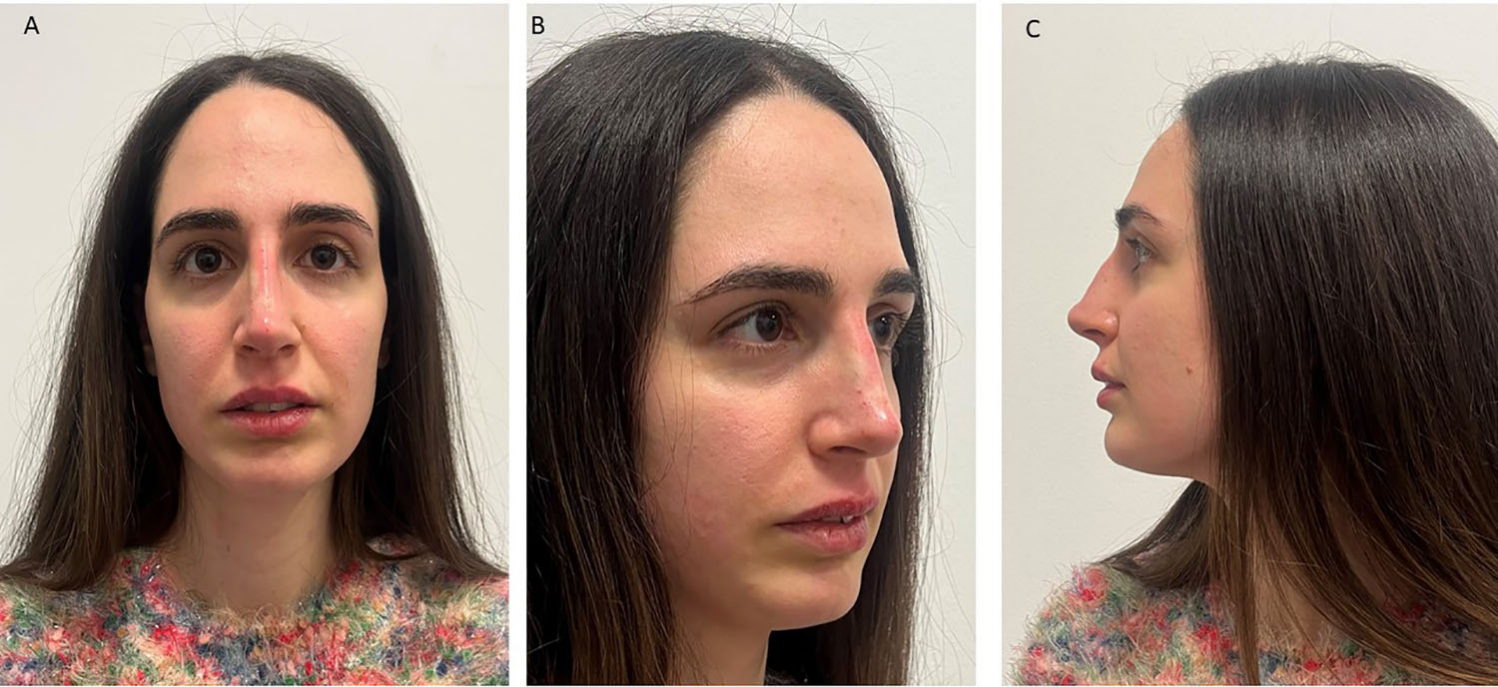
Immediately after HA infiltration shows an improve the projection of the tip of nose in frontal view (**A**), lateral view (**B**), and at 45 degrees (**C**)

**Fig. 8:**
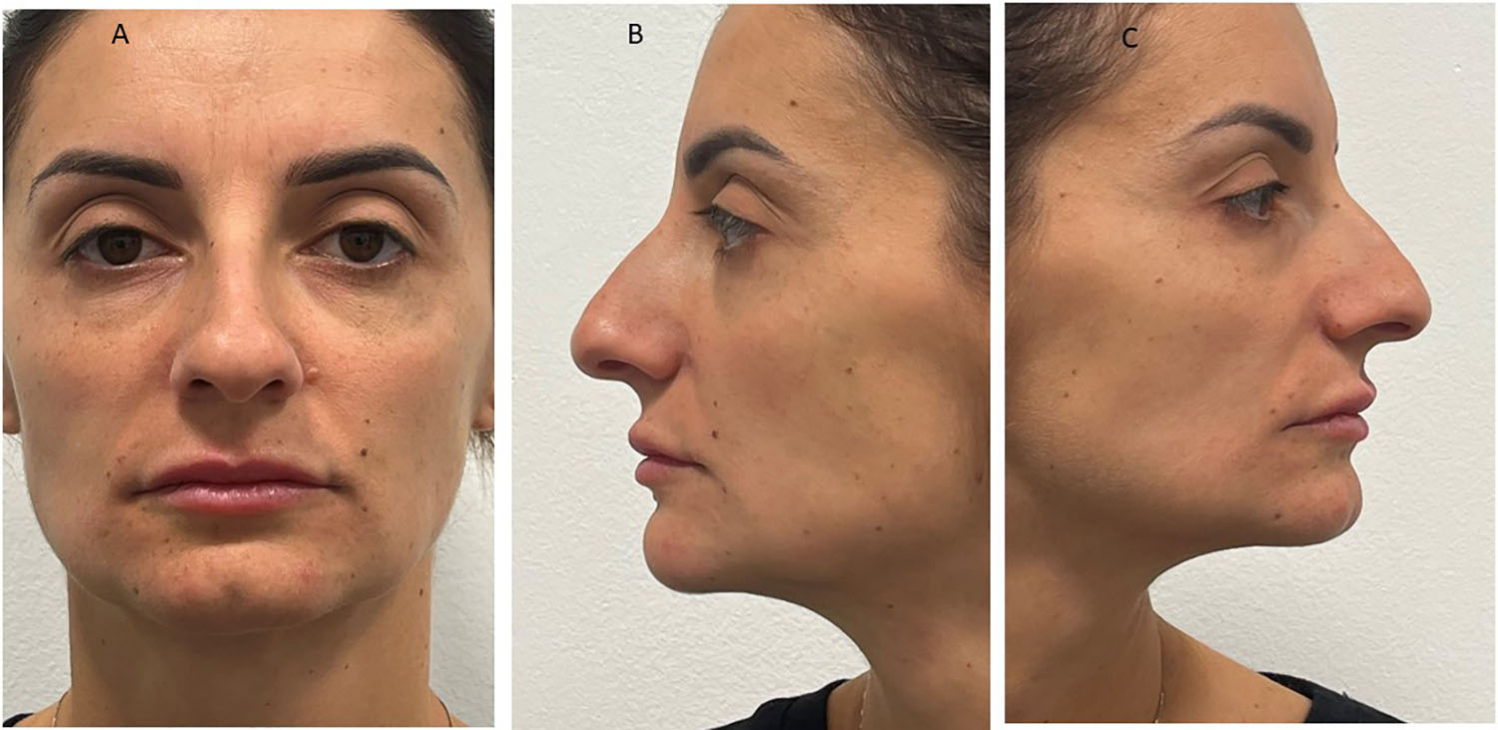
The initial state of pre-treatment. The patient shows a hypo-projected tip in frontal view (**A**), lateral view (**B**), and at 45 degrees (**C**)

**Fig. 9:**
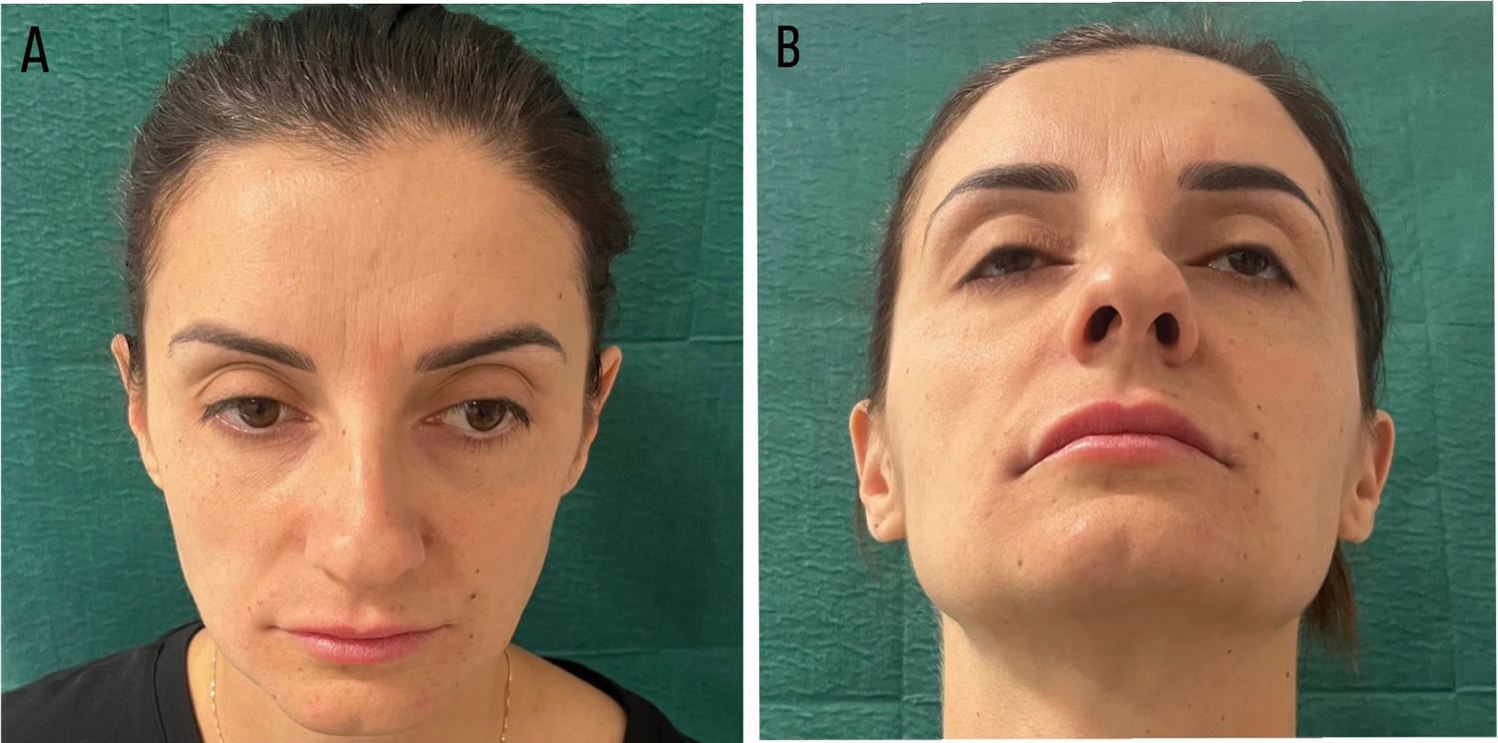
The initial state of pre-treatment. Head tilted forward (**A**) and back (**B**) view of the face over the shoulder

**Fig. 10:**
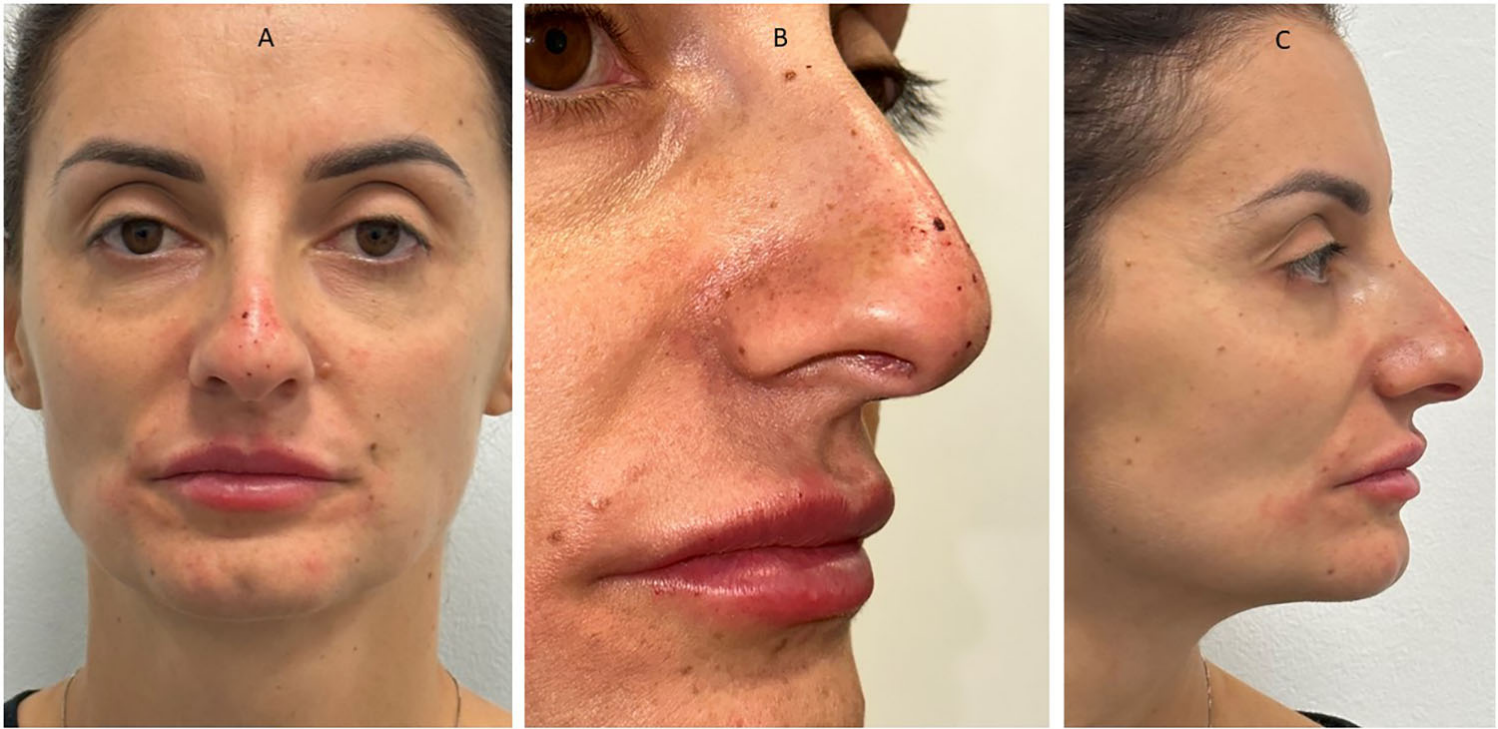
Immediately after HA infiltration shows a improve the projection of the tip of nose in frontal view (**A**), lateral view (**B**), and at 45 degrees (**C**)

**Fig. 11:**
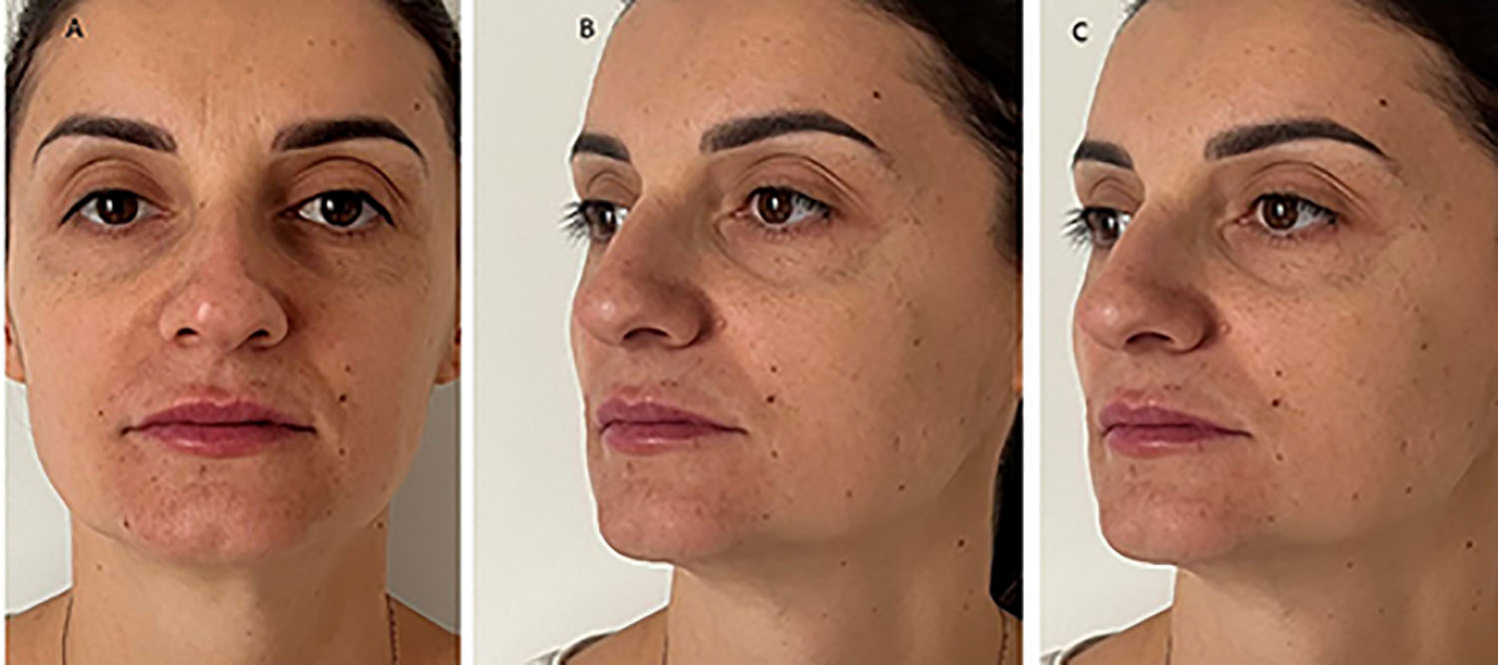
View of the nose after 180 days after of rhinofiller infiltration shows a improve the projection of the tip of nose in frontal view (**A**), lateral view (**B**), and at 45 degrees (**C**)

**Fig. 12:**
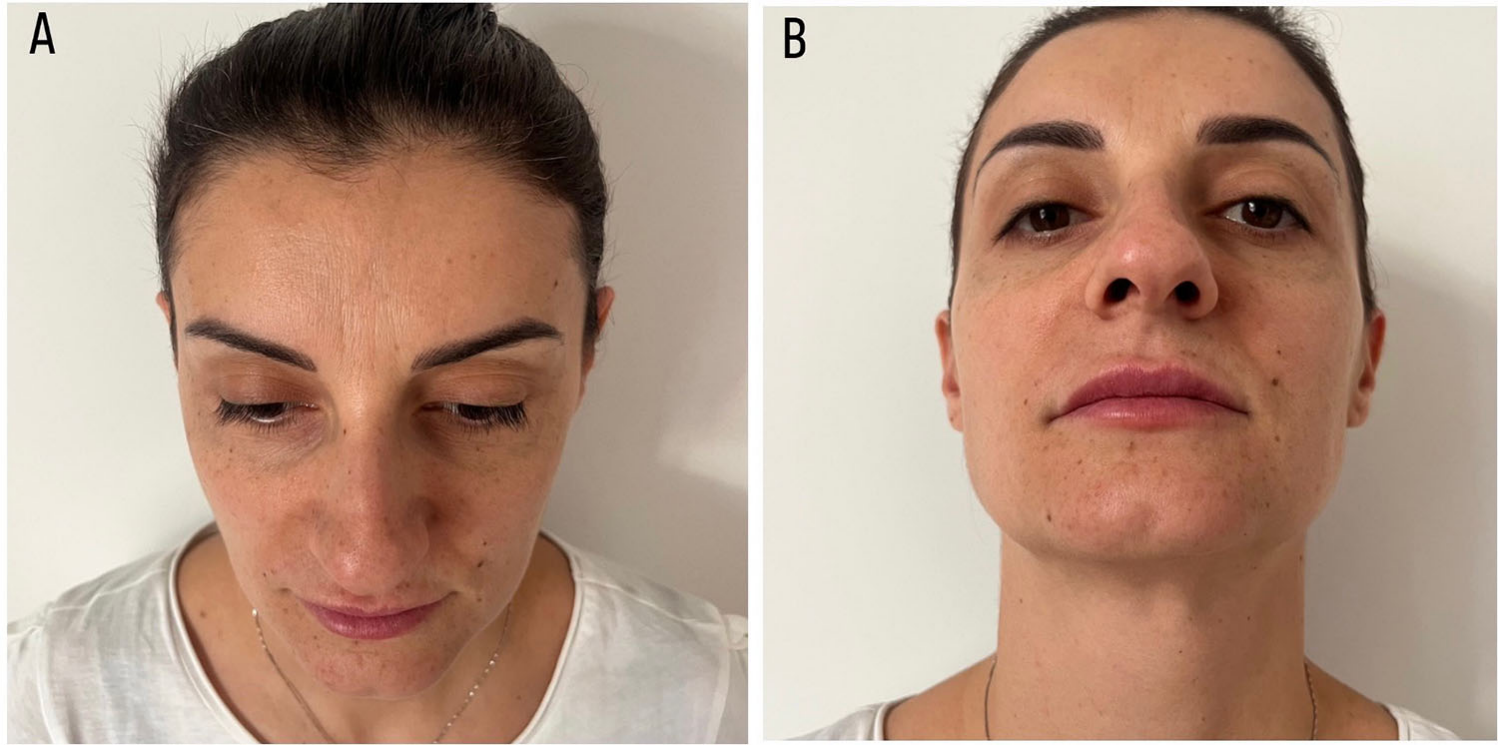
View of the nose after 180 days after of rhinofiller infiltration. Head tilted forward (**A**) and back (**B**) view of the face over the shoulder

A total of 420 sample images have been evaluated for the anthropometric assessment. The inter-examiner reliability of the anthropometric measurements was consistently high, with a Cohen’s kappa (κ) coefficient of 0.81 (*p*<0.01). The Bland–Altman plot is presented in Fig. 5. A total of 1260 measurements have been considered for the anthropometric assessment, and the data output is summarized in Table 1. The NFrA means were at T_0_, T_90_, and T_180_, respectively, 147.1±2.13, 141.9±3.30, and 142.6±2.13. A significant difference NFrA was detected comparing the T_0_ and T_90_ (*p*<0,0001), while no difference was present comparing T_90_ and T_180_ means (*p*=0.11) (Table 1). The NFaA means were at T_0_, T_90_, and T_180_, respectively, 22.05±1.17, 25.98±1.55, and 23.32±2.90 (Fig. 6). A significant difference NFaA was reported comparing the T_0_ and T_90_ (*p*<0,0001), and comparing T_90_ and T_180_ means (*p*<0,0001) (Table 1). The NLA means were at T_0_, T_90_, and T_180_, respectively, 92.05±2.83, 106.4±2.49, and 98.51±10.44 (Figs. 7, 8, and 9). A significant difference NFaA was detected comparing the T_0_ and T_90_ (*p*<0,0001), and comparing T_90_ and T_180_ means (*p*<0,0001) (Table 1 and Figs. 6, 7, 8, 9, 10, 11, and 12).

**Table 1:** Measurements considered for the anthropometric assessment and the data output

	[NFrA]			[NFaA]			[NLA]		
	T_0_	T_90_	T_180_	T_0_	T_90_	T_180_	T_0_	T_90_	T_180_
Mean	147.1	141.9	142.3	22.05	25.98	23.32	92.05	106.4	98.51
Std. deviation	2.13	3.30	4.25	1.17	1.55	2.90	2.83	2.49	10.44
Std. error of mean	0.18	0.27	0.35	0.09	0.13	0.245	0.23	0.21	0.88
Lower 95% CI	146.8	140.5	141.9	21.85	25.72	22.84	91.58	105.9	96.77
Upper 95% CI	147.5	141.6	143.3	22.25	26.24	23.81	92.52	106.8	100.3
Minimum	142.5	135.4	136.4	19.49	21.75	18.25	85.7	100.4	84.18
Maximum	152.3	147.2	147.8	25.92	28.91	29.01	97.02	110.3	111.6
Range	9.745	11.82	11.4	6.435	7.165	10.76	11.32	9.91	27.43

## Discussion

The outcome of the present clinical study gives greater projection and upper rotation to the tip with great gratification of the patients and the surgeon. An augmentation of the tip nose with hyaluronic acid filler produces a rejuvenation of the nose area resulted in a more youthful appearance. The infiltrative bolus technique used consent to improving the appearance of the tip of the nose with a safety and simple procedure without incurring in complication [16]. The results showed persistent until 6 months. Tip nose augmentation with HA has been widely utilized to rejuvenate nose. Rhinofiller called also nasal augmentation, medical rhinoplasty, liquid rhinoplasty, non-surgical rhinoplasty, and injection rhinoplasty is used to describe the technique of injecting fillers into the nasal pyramid region for aesthetic, and occasionally functional, purposes. However, the terms rhinofiller is widely used and usually was used a needle or cannula [17]. The filler used for the correction of the nose, also called rhinofiller, is particularly suitable in particular patients, such as the following:

1. Patients who do not want to undergo or are unable surgery for concomitant a diseases that contraindicate surgery [18]
2. Patients who do not desire major changes of aged nose
3. Patients who have already undergone rhinoplasty surgery [18] and have need only minor correction

Many authors prefer to filling well-established areas such as nasal radix, nasal dorsum, nasal tip, and supratip [19]. Other authors prefer an injection technique that starts at the nasal tip, and continues with columellar base, dorsum, and nasal root [20]. This preference devotes the first attention to reshaping the caudal part of the nose. Increased nasal projection may also contribute to a thinner appearance of the tip of the nose. This operation results in secondary stretching of the soft tissues and nostrils in caudal view, thus achieving a narrowing of the width of the nose from one wing margin to the other in frontal view. Sometimes the injection sequence may depend on the use of needle-cannulas as opposed to needles because the introduction of the cannula requires a pathway with retrograde inoculation, so it is preferred to inoculate the more distal areas and then release the filler backward towards the exit hole, which is usually represented by the nasal tip.

In fact, the use of needle-cannulas involves an entrance hole located at the tip and two working areas that follow the nasal profile in the two directions.

Most authors inject in the dome area, in the so-called weak converse triangle. Filling this area results in an increased projection of the nasal tip. This area and the inter-columellar area are the ones treated in the Italian technique descript in the present paper.

The retro-columellar area is filled to determine support for the tip structure and to achieve upward rotation of the tip. It is critical to maintain control of the filler in the median position to avoid obstruction of either nostril due to accidental lateral projection of the material. Treatment of the inter-columellar area also improves the projection of the columella when it is insufficient and together with the treatment of the nasal spine, contributes to the opening of the nasolabial angle, improving the appearance of this district. From a safety point of view, it is fundamental to make the following considerations. The use of fillers may still occasionally lead to complications caused by intravascular injecton, such as necrosis and embolism. In order to avoid those adverse events, we choose these two sites (fibrous septum and the interdomal space) because they are very safe, free from important vassels. The subnasal artery runs together with the lateral accessory labial artery, along the filter and the columella above the floor outside of the same muscle. Fibrous septum is posterior whereas interdomal space is deeper to the vascular network. Rare adverse events described in the literature after filler treatments, such as seroma, organized haematoma, migration of filler, fibrosis, stains, infections, allergic reactions, impaired muscle function, inflammatory reactions, nodules/abscess, granulomas, dysesthesia/paraesthesia/anaesthesia, persistent scarring, tissue necrosis, and embolism did not occur.

Rhinofiller, called also non-surgical rhinoplasty, is a great possibility for patients that have little deformities of their nose, such as a dorsal hump or little asymmetries. Rhinofiller is also used to correct small deformities after rhinoplasty. This is a good technique especially in old patient that requesting minimally invasive treatment with little or no downtime and reversible. Limitation of HA fillers treatment for rhinoplasty: HA degrades over time (short duration of the effect). A recent systematic reviews and meta-analyses paper by Beneduce et al. [21] descripted that rhinofiller produces a longevity results until 8 years. From this review, it is impossible establishing with certainty the longevity of non-surgical rhinoplasty because it is extremely complex depend of HA type used. Many authors descripted that the result remains stable at 4 and 8 months, requiring 1–2 injection sessions touch-ups in the 1st year and 1 per year during the following years [22]. The rhinofiller is a safety technique but a low percentage of complication has been descripted such as bruising, haematoma, infection, skin necrosis, and vision loss [23]. A recent retrospective review of 5000 treatments shows that the rhinofiller is a safe procedure with positive aesthetic results with little complication [24]. The percentage of complication outcome across all cohort studies was 2.52%. The most commonly reported complications were haematoma (0.13%) and bruising (1.58%). While uncommon, there are several reports of most important complications including 7 reports of skin necrosis (0.08%), 30 episodes of vessel occlusion (0.35%), 8 reports of vision loss (0.09%), and 6 reports of infection (0.07%) [25]. Rhinoplasty using HA fillers allows to maintain the heigh of the nose, which sometimes can be difficult to achieve with a surgical procedure.

Moreover, nasal reshaping with HA has been proposed not only as an alternative for the cosmetic field but also as a complement for nose surgical procedures [13]. The use of injectable HA offers many and important advantages, including improvements in skin texture and quality, low morbidity, rapid recovery, and low cost. Furthermore, the study has been reported no major adverse events occurred after the procedure. This testifies the high benefit–risk ratio attributable in part to the technique and in part to the gel used, both in terms of concentration and rheological characteristics and quality of the cross-linked hyaluronic acid.

Usually, elective procedure for younger patients is rhinoplasty, however, in the older patients are required a non-surgery rhinoplasty with dermal filler for aesthetic indications.

It is essential to note that this technique has limitations, such as the fact that it does not change the structure of cartilage and bone and cannot reduce but only add, which requires repeated infiltrations in the time.

In conclusion, the Italian technique descripted in the present paper is safe, simply, and efficacious for rejuvenation of the nose, with elevated levels of patient satisfaction. It has capability to be an important tool in non-surgical rhinoplasty. It is knowledge of nasal anatomy, and use of appropriate materials and technique are key in ensuring safety and results. The Italian technique differs from other non-surgical methods because they use two very safe sites (fibrous septum and the interdomal space) because they are free from important vessels also allowing a good aesthetic result.
